# 

**Published:** 1859-02

**Authors:** 


### CONTENTS.

ORIGINAL COMMUNICATIONS— PAGE.

Correct Use of Alkalies and Acids in Practice. By E. Andrews, M.D... 69

Do Inflammations require a Different Treatment from that pursued Fifteen or Twenty Years ago? By B. S. Woodworth, M.D.................. 80

An Enquiry, as to Whether and How "Enemata Nutrientiæ" are Absorbed from the Rectum, so as to prove Nutrient to the System. By B. Woodward, M.D.................................................. 86

Surgical Cases. By C. Brackett, M.D........................................ 89

Fractures of the Lower Jaw. By O. C. Gibbs, M.D........................ 90

Ligation of the Tibilias Anticus Artery. By A. Heavenridge, M.D...... 91

PROCEEDINGS OF MEDICAL SOCIETIES—

Rock River Union Medical Society........................................ 93

Shelby County Medical Society............................................ 99

Second Meeting of the Champaign County Medical Society.................. 101

Nashville Medical Society.................................................. 114

BOOK AND PAMPHLET NOTICES—

A Treatise on Human Physiology.......................................... 103

The Science and Art of Surgery............................................ 103

A Practical Treatise on the Diseases of Children......................... 104

The Functions of the Cerebellum.......................................... 104

ABSTRACTS AND ITEMS. Prepared by the Junior Editor...................... 106

EXTRACTS........................................................................ 108

EDITORIAL—

Annual Commencement...................................................... 115

Summer Course of Medical Instruction..................................... 117

Preliminary Education, etc................................................ 118

American Medical Association.............................................. 120

Resignation...................................................................... 122

CORRESPONDENCE.............................................................. 122

